# *Carlavirus* Species Infecting Hop Plants in Italy: Molecular Identification and Phylogenetic Analyses of the Detected Isolates

**DOI:** 10.3390/plants12193514

**Published:** 2023-10-09

**Authors:** Marta Luigi, Livia Donati, Renato Sciarroni, Andrea Gentili, Anna Taglienti, Antonio Tiberini, Francesco Faggioli, Luca Ferretti

**Affiliations:** Council for Agricultural Research and Economics (CREA), Research Centre for Plant protection and Certification, Via C.G. Bertero, 22, 00156 Rome, Italy; marta.luigi@crea.gov.it (M.L.); livia.donati@crea.gov.it (L.D.); sciarroni.renato@crea.gov.it (R.S.); andrea.gentili@crea.gov.it (A.G.); anna.taglienti@crea.gov.it (A.T.); antonio.tiberini@crea.gov.it (A.T.); francesco.faggioli@crea.gov.it (F.F.)

**Keywords:** hop carlaviruses, detection, RT-PCR, sequencing, Italy

## Abstract

Hop (*Humulus lupulus* L.) is a minor ingredient in the beer production but has a strong influence on the beer quality due to the high chemical complexity of the cones used in brewing. One of the major factors that can severely affect the chemical composition of the hop cones and their marketability is the presence of viral infections in the plant. Amongst the five major hop viruses, three belong to the *Carlavirus* genus: hop mosaic virus (HpMV), hop latent virus (HpLV), and American hop latent virus (AHLV). The occurrence of carlaviruses on hop germplasm in Italy was firstly recorded in 2017 but, in that context, a generic detection was only performed and no information on the infecting *Carlavirus* species was provided. To fill this gap, 51 hop samples previously found infected by carlaviruses were analysed by RT-PCR employing primer pairs specific for the coat protein (CP) of HpMV, HpLV and AHLV, respectively. HpLV resulted largely prevalent as it was detected in 96.1% of tested samples whereas for HpMV and AHLV an infection rate of 5.9% and 3.9% was recorded, respectively. CP nucleotide sequences from 13 selected virus isolates were obtained and analysed; moreover, the complete genome sequence of 7 isolates was obtained by using high throughput sequencing (HTS). Phylogenetic analysis showed close relationships among isolates from different geographical origin, including European and non-European countries, according to the worldwide movement of hop germplasm due to global trade. This is the first report of HpMV, HpLV and AHLV on hop germplasm in Italy.

## 1. Introduction

Hop (*Humulus lupulus* L.) belongs to the genus *Humulusis* consisting of dioecious, perennial, climbing vines. This genus belongs to the *Cannabaceae* family of the *Urticales* order incorporated into the natural order of *Rosales*. Hop is present throughout the temperate climate regions (between latitude 35° and 55°) in both the Hemispheres, mainly cultivated for female flowers, known as cones, which are endowed with a complex chemical composition accounting for more than 300 chemical species including resins, essential oils, polyphenols lipids, waxes, cellulose, and amino acids [1]. These components are used in brewing industry to impart bitterness, aroma, and flavour to beer, contribute to its microbial stability, and enhance and stabilize its foam. Hence, though being a minor ingredient in the beer production, hop has a strong influence on the beer quality.

In recent years, the use of hop was not limited to the brewery sector; in fact, the wide chemical composition of hop was exploited also for pharmaceutical applications due to anti-inflammatory, antimicrobial, antioxidant and antiproliferative properties, its effects on glucose metabolism, on hormone regulation, lipid management and sedative/hypnotic capacity [2].

One of the major factors that can severely alter the chemical composition of the hop cones, compromising their marketability, is the presence of viral infections in the plant [3]. The main viral pathogens affecting hop include five viruses and two viroids [4], worldwide distributed and able to cause important losses in commercial hop yards. In addition, a range of minor diseases, caused by both viruses and viroids, were described with low incidence or in localized regions [4]. 

Amongst the five major hop viruses, three belong to the *Carlavirus* genus (family: *Betaflexiviridae*): hop mosaic virus (HpMV) [5,6], hop latent virus (HpLV) [7,8], and American hop latent virus (AHLV) [9,10]. 

HpMV was associated to the hop mosaic disease, firstly, described in United Kingdom by Salmon (1923) in association with clearly leaf symptoms that, on the susceptible cultivars, could bring to a lethal reaction [11]. HpMV was then reported worldwide, affecting hop in all the continents. Luckily, modern cultivars are generally tolerant to HpMV infection and do not exhibit any symptom; only some old mosaic-susceptible cultivars can develop a typical clearing or yellow banding along main veins in addition to stunting and reduction in the number of cones [4]. 

HpLV was reported for the first time by Schmidt et al. [12] and, as HpMV, it has been successively reported in hop yards all over the word: Europe [7,13], the United States [10], New Zealand [14], Australia [15,16], China [17], South Africa [18] and Japan [19]. 

AHLV is common only in the United States, although with a lower frequency with respect to the other carlaviruses of hop [10], but it was reported sporadically also in New Zealand [20]. In Germany [21], the United Kingdom [21] and Australia [15] it was detected in post-entry quarantine of hop breeding material and promptly eradicated.

Virions of these three viruses show common characteristics that are generally investigated to molecularly characterized isolates belonging to each species. They are filamentous and flexuous particles, and their genomes are single stranded monopartite RNAs of about 8.5 kb in length, organized in 6 different open reading frames (ORF). ORF5, encompassing the putative coat protein (CP), is characterized by two highly conserved regions encoding for two distinctive segments of the CP. The first is located in the core region of the protein (according to Hillman and Lawrence [22]), and it results conserved also in the CP of potexviruses [23] while the second is located near the C-terminal region [24].

HpMV, HpLM and AHLV are demonstrated to be transmitted in a non-persistent manner by the aphid vector *Phorodon humuli* [5]; for HpMV and HpLV, transmission by other commonly spread aphid species such as the green peach aphid *Myzus persicae* and the potato aphid *Macrosiphum euphorbiae* has been ascertained [25]. Presence of *P. humuli* and aphids belonging to the *Macrosiphum* genus has been recently recorded in some Italian hop yards [26]. 

It is quite difficult to assess the effect of carlaviruses on hop growth and cones yield and quality due to the multiple virus and viroid infections that generally affect hop [4]. However, a decreasing in alpha and beta acids level and their ratio, together with a reduction of lateral length, leaf weight, and the number of nodes per lateral have been reported by several authors [3,14,27].

The occurrence of viruses belonging to the *Carlavirus* genus in hop germplasm in Italy was firstly recorded in 2017 in the frame of a field monitoring programme aimed at evaluating the phytosanitary status of Italian hop yards [26]. In that context, a generic detection based on the use of carlavirus genus-specific PCR primers was performed, and no information was yet available on the *Carlavirus* species infecting hop in Italy. To fill this gap, hop samples previously found latently infected by carlaviruses were further investigated in order to identify the presence of HpMV, HpLV and AHLV and to explore the molecular features and variability of the detected virus isolates. To our knowledge, this work represents the first report on the occurrence of HpMV, HpLV and AHLV on hop germplasm in Italy. 

## 2. Results

### 2.1. Identification of Carlavirus Species 

According to the primer pair used, amplification bands of the expected sizes were obtained from all the RNA extracts analyzed by RT-PCR amplification. Specifically, out of 51 carlaviruses-infected samples tested, 49 (96.1%) gave positive reaction with the primers specific for HpLV, 3 (5.9%) with the primers specific for HpMV (including the wild hop sample from Emilia-Romagna region) and 2 (3.9%) with the primers specific for AHLV (Table 1). Three samples showed a mixed infection; specifically, two samples from Emilia-Romagna region (FZ1 and FZ3) resulted positive for HpLV and AHLV whereas one sample from Tuscany (CEN2) was found infected by HpLV and HpMV.

### 2.2. Nucleotide Sequence and Phylogenetic Analyses of ORF5s (CP)

Overall, twelve complete (HpMV and HpLV) and one partial (AHLV) CP sequences from selected isolates were deposited in the NCBI database. GenBank accession numbers are listed in Table 1.

**Hop latent virus**. Out of 49 HpLV isolates identified, 9 isolates (Abr1, FZ3, FZ5, H1, OB1, RI6, RN2, TDF3, W2) from hop plants belonging to different cultivars and of different geographical origin were selected to be sequenced.

**Figure 1:**
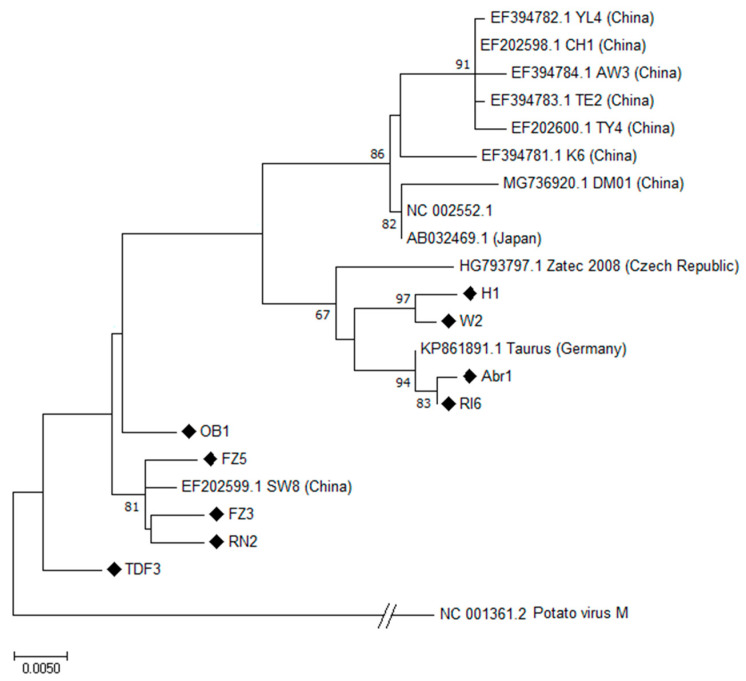
Phylogenetic analysis of the CP gene of hop latent virus (HpLV) by Maximum Likelihood method based on the Kimura 2-parameter model [28] with bootstraps of 1000 replications. The percentage of trees in which the associated taxa clustered together (>50) is shown next to the branches. The CP nucleotide reference sequence of potato virus M was included in the analysis as outgroup. (♦) indicates the Italian isolates.

The complete ORF5 nucleotide sequence was obtained for all the selected isolates. The obtained sequences shared an identity percentage ranging from 95.61 to 99.59% among themselves. From BLAST analysis, isolates FZ3, FZ5, OB1 and RN2 showed the highest sequence identity (from 98.70 to 99.13%) with the isolate SW8 (EF202599.1) from China, whereas an identity ranging from 96.22 to 99.59% with the German isolate Taurus (KP861891.1) was observed for the isolates Abr1, H1, RI6, TDF3 and W2. According to the sequence data, phylogenetic analyses showed that the nine HpLV isolates from Italian hop yards grouped into two distinct and highly separated clusters of the phylogenetic tree (Figure 1).

**Hop mosaic virus**. Complete ORF5 nucleotide sequence of HpMV was obtained from isolates RN1, CEN1 and CEN2. 

Isolates CEN1 and CEN2, both collected from the same hop yard, showed 100% sequence identity among themselves, whereas they shared an identity percentage of 95.3% with the isolate RN1 from a wild hop. From BLAST analyses, the three sequenced isolates showed the highest identity with the CP gene of the AUS-FR-2A isolate (FJ463804.1) from Australia, in a range from 95.90 (CEN1 and CEN2) to 99.00% (RN1).

**Figure 2:**
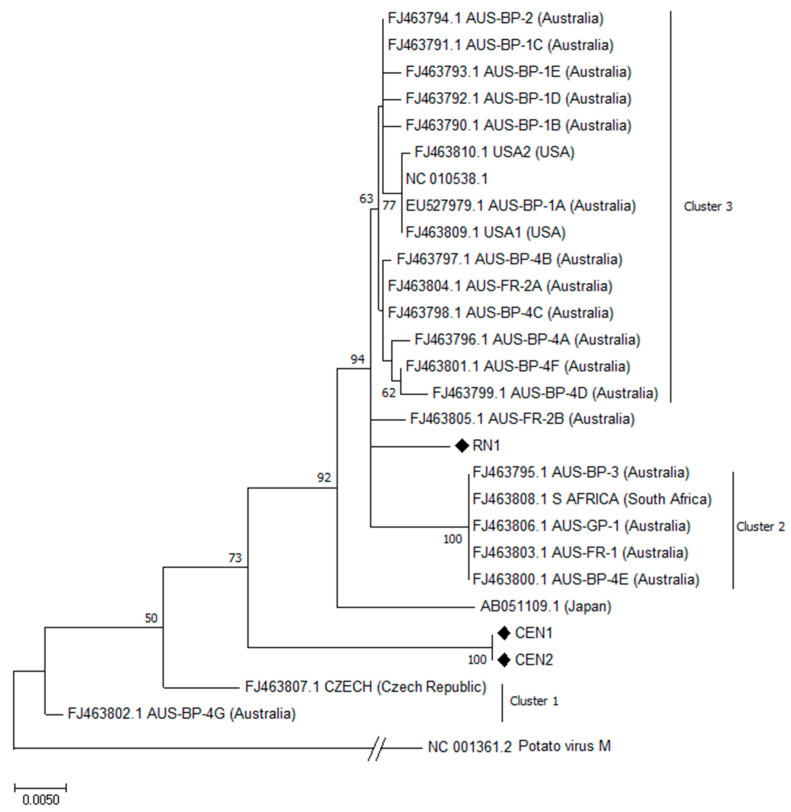
Phylogenetic analysis of the CP gene of hop mosaic virus (HpMV) by Maximum Likelihood method based on the Kimura 2-parameter model [28] with bootstraps of 1000 replications. The percentage of trees in which the associated taxa clustered together (>50) is shown next to the branches. The CP nucleotide reference sequence of potato virus M was included in the analysis as outgroup. The three annotated clusters refer to the phylogenetic structure reported in Poke et al. [29]. (♦) indicates the Italian isolates.

From the phylogenetic analysis (Figure 2), none of the sequenced isolates grouped into one of the three putative clusters identified [29]; the RN1 isolate from wild hop resulted more closely related to the ungrouped Australian isolate AUS-FR-2B, identified in the commercial hop cultivar Nugget, whereas isolates CEN1 and CEN2 grouped together in a separate branch. 

**American hop latent virus**. A partial CP nucleotide sequence was obtained for one (FZ3) out of two AHLV isolates identified by specific RT-PCR amplification. 

Comparisons with sequences from the NCBI database showed the highest identity (98.22%) with the isolate 23VIC (KF749273.1) from Australia. 

**Figure 3:**
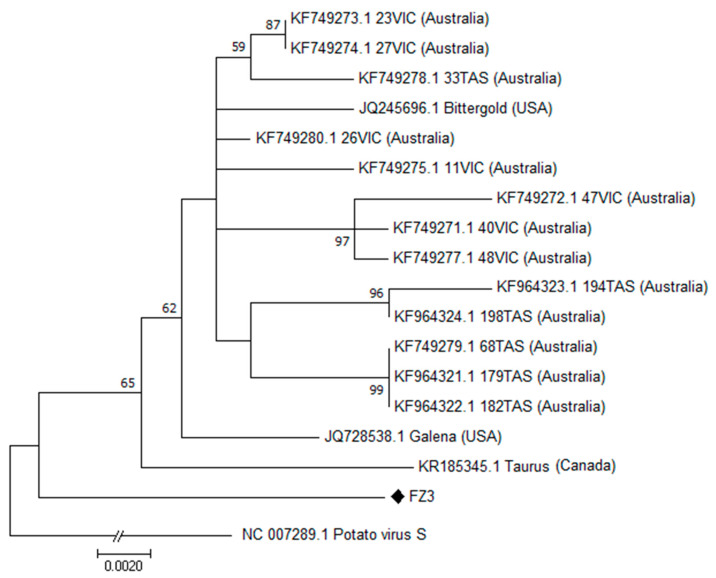
Phylogenetic analysis of the CP gene of American hop latent virus (AHLV) by Maximum Likelihood method based on the Kimura 2-parameter model [28] with bootstraps of 1000 replications. The percentage of trees in which the associated taxa clustered together (>50) is shown next to the branches. The CP nucleotide reference sequence of potato virus S was included in the analysis as outgroup. (♦) indicates the Italian isolate.

The phylogenetic analysis (Figure 3) showed that the FZ3 isolate clustered in a separate branch of the phylogenetic tree, close to the ungrouped isolates Galena (JQ728538) from USA and Taurus (KR185345) from Canada.

### 2.3. Virome Analysis and Viral Genomes Assembly

Analysis of the virome composition performed using GAIA software confirmed the presence of the expected viruses in the sequenced samples. Overall, seven complete genome sequences from selected hop carlavirus isolates were assembled and deposited in the NCBI database: one AHLV (FZ3 isolate), four HpLV (CEN2, R16, FZ3 and FZ5 isolates) and two HpMV (CEN2 and RN1 isolates). GenBank accession numbers are listed in Table 1. For all the assembled genomes the six constitutive ORFs were retrieved and neither insertions nor premature stop codons were found. The nucleotide and aminoacidic sequence of each ORF was compared with the sequences available in the NCBI database (Table 2). Overall, homologies at nucleotide level ranged from a minimum of 93.58% (ORF3, HpMV isolate CEN2) to 100% (ORF4, HpMV isolate RN1), whereas for the aminoacidic sequences they were generally higher (from 96.99 to 100%), except for the ORF4 of the HpMV isolate CEN2 for which a value of 92.65% was recorded. This latter isolate resulted the most divergent, showing the lowest values of nucleotide identity for the ORF1 (93.81%), ORF2 (94.78%), ORF3 (93.58%), ORF4 (93.72%) and ORF5 (95.56%). Conversely, the AHLV genome from FZ3 isolate showed a very high identity percentage with the currently know isolates for all the six ORFs, at both the nucleotide and aminoacidic levels. With specific regard to the CP genomic region, the complete ORF5-nucleotide sequence of AHLV isolate FZ3 resulted 98.07% identical to that of Galena isolate (JQ728538) from USA, providing further evidence of a phylogenetic relationship between these two isolates preliminarily observed by phylogenetic analysis of the partial CP-nucleotide sequence generated from the RT-PCR amplicon. Among the four HpLV assembled genomes, RI6 isolate resulted the most conserved showing high identity percentage with sequences available in the NCBI database for all the ORFs, at both the nucleotide and aminoacidic levels. The other HpLV isolates (FZ3, FZ5 and CEN2) showed a higher degree of molecular variability, especially on ORF1, ORF2 and ORF3. 

**Figure 4:**
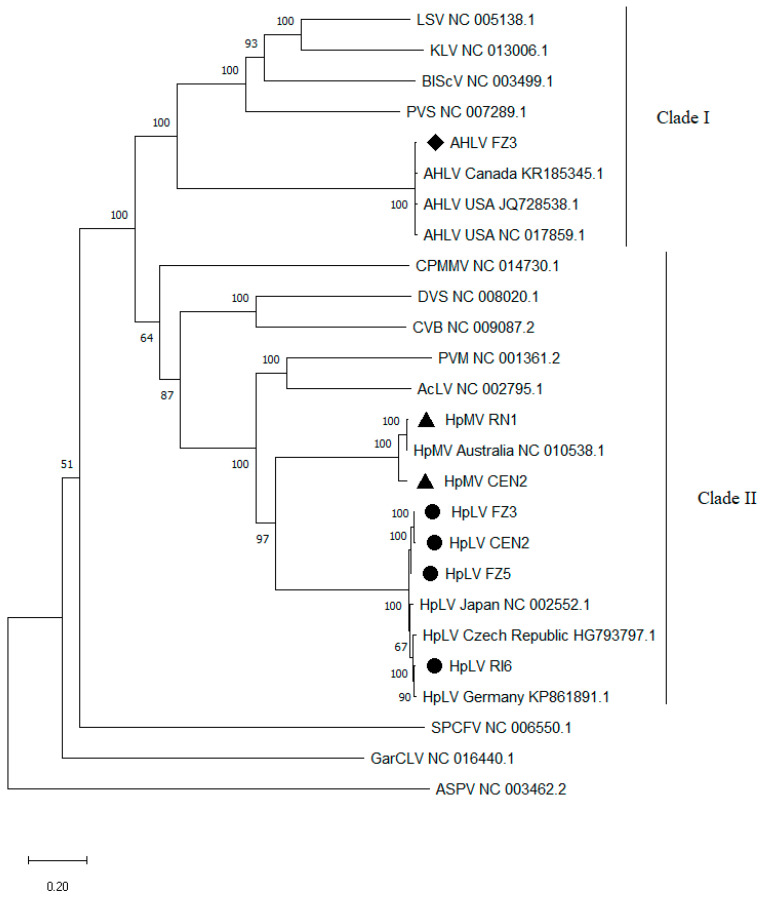
Phylogenetic analysis of the complete genomes of different *Carlavirus* species by Maximum Likelihood method based on General Time Reversible model [30]. Reference genomes of the species: aconitum latent virus (AcLV); blueberry scorch virus (BlScV); chrysanthemum virus B (CVB); daphne virus S (DVS); cowpea mild mottle virus (CpMMV); garlic common latent virus (GarCLV); kalanchoe latent virus (KLV); lily symptomless virus (LSV); potato virus M (PVM); potato virus S (PVS) and sweet potato chlorotic fleck virus (SPCFV) belonging to the different carlavirus clades [31] were retrieved from NCBI. For AHLV, HpLV and HpMV all the complete genomes present in the NCBI database were included and their origin was reported. The branches marked with ♦, ● and ▲ indicate the complete genomes obtained from the Italian isolates of AHLV, HpLV and HpMV, respectively. The percentage of trees in which the associated taxa clustered together (>50) is shown next to the branches. The reference genome of apple stem pitting virus (ASPV) was included in the analysis as outgroup.

From the phylogenetic analysis carried out employing all the complete genomes of carlaviruses currently available on the NCBI database (Figure 4), AHLV genomes clustered in a distinct clade (referred as clade I) including carlaviruses from different host plant species (lily symptomless virus—LSV, kalanchoe latent virus—KLV, blueberry scorch virus—BlScV, potato virus S—PVS), distantly related from that including HpMV and HpLV genomes (referred as clade II). Within the clade I, AHLV isolates grouped all together in a distinct branch, showing a very low degree of molecular variability among themselves. A closer phylogenetic relatedness was found among the genomes of HpMV and HpLM isolates that clustered separately on two distinct but close branches of the phylogenetic tree, within the clade II.

## 3. Discussion 

Among hop viruses, the *Carlavirus* species HpMV, HpLV and AHLV are considered major pathogens on hop. While HpMV and HpLV are known to occur worldwide, a more restricted distribution area is reported for AHLV, essentially present in North America. Molecular investigations carried out on carlavirus-infected samples collected from different Italian hop yards revealed the presence of HpMV, HpLV and AHLV in hop plants belonging to different commercial cultivars. Identification was performed by conventional RT-PCR amplification employing specific primer pairs targeted to the CP gene of each virus. For HpMV and HpLV, primers were purposedly designed in the present work. Sequencing of the generated amplicons and BLAST analysis confirmed the specificity of the employed primer pairs previously ascertained by in silico analysis. HpLV resulted largely prevalent as it was detected in 96.1% of carlaviruses-infected samples tested. Conversely, a low infection rate was recorded for HpMV (5.9%) and AHLV (3.9%), the latter always in mixed infection with HpLV. All analysed samples did not show any symptom referable to possible infections by carlaviruses, according to their generally latent status. To the best of our knowledge, this is the first report of HpMV, HpLV and AHLV on hop in Italy.

For all the identified virus isolates, nucleotide sequence data and phylogenetic analysis of the CP gene showed close relationships with isolates from different geographical origin, including European (Germany) and non-European (Australia, Canada, USA, China) countries, according to the world-wide movement of the hop germplasm. The occurrence of these close relationships was also confirmed by analysis of the complete genomes obtained by HTS. 

Evidence supporting the hypothesis of a possible introduction of hop viruses by planting material were primarily found in case of the two samples found infected by AHLV (FZ1 and FZ3 isolates): they were both from the same hop yard established with rhizomes purchased on the web from an American seller and exhibited the same infection pattern (HpLV + AHLV). Sequencing of the complete genome of FZ3 isolate provided molecular evidence to this hypothesis highlighting a nearly 100% sequence identity among FZ3 and isolates from Canada and USA, according to the present distribution of AHLV in the world. Similar evidence was obtained from the HpLV isolates H1, RI6, Abr1 detected in plants imported from Germany, for which high sequence similarity (98–99%) and close phylogenetic relationships with the German isolate ‘Taurus’ were recorded at the CP level. This was further confirmed at the genome level for the RI6 isolate for which all the ORFs exhibited the highest identity percentage with the nucleotide sequences of the Taurus isolate from Germany. 

Phylogenetic analysis carried out on CP of HpMV isolates pointed out the three putative clusters previously described by Poke et al. [29]. According to this phylogenetic structure, HpMV isolates from Italian hop yards resulted ungrouped as the two isolates from Japan (AB051109) and Australia (FJ463805). Interestingly, the CP gene of the RN1 isolate detected in a wild hop collected from an area surrounding a commercial hop yard showed high sequence similarity (99%) with that of the Australian HpMV isolate AUS-FR-2A (FJ463804) detected in the hop cultivar Agate, and no specific traits referable to a possible wild type were found. High molecular similarities and close phylogenetic relationships with Australian isolates identified in cultivated cultivars were confirmed when the complete genome sequence of the HpMV isolate RN1 was analysed and compared with the available ones, opening questions on the role of wild hop as reservoir/final host in the natural movement of the virus in the agricultural ecosystem.

Finally, the phylogenetic analyses carried out on the complete genome sequences of carlaviruses currently available confirmed the phylogenetic position of HpMV and HpLV isolates reported in other papers [32], indicating a close relatedness between these two hop virus species, while a divergent position was ascertained for the AHLV isolates. 

The results reported in the present paper reinforce the need of performing specific diagnostic tests for such viruses (AHLV, HpLV and HpMV), especially on nuclear stocks of plant propagation material, in order to avoid their spreading by vegetative multiplication, commonly adopted in hop cropping. With this regard, information collected from the growers on the planting material used for establishing the hop yards evidenced differences in the geographical origin of plantlets and rhizomes and in the modalities of supplying, also including the web market of rhizomes. In this scenario and in the absence of phytosanitary regulations covering these viruses, their circulation in the plant propagation material appears to be greatly promoted.

## 4. Materials and Methods

### 4.1. Source of Plant Material

A total of 51 asymptomatic leaf samples collected in 2017 from cultivated (50) and wild (1) hop plants previously found infected by *Carlavirus* genus viruses [26] were submitted to molecular analyses aimed at identifying and characterizing the infecting specie. Cultivated hop plants sampled belonged to different international cultivars and came from different commercial hop yards located in northern (Emilia-Romagna) and central (Lazio, Tuscany, Abruzzo, and Marche) Italian regions. The wild hop sample was collected from an area surrounding cultivated hops located in Emilia-Romagna region. Origin, number, and cultivar of the analysed samples are listed in Table 3.

### 4.2. Total RNA Extraction

Total RNA was extracted from plant samples previously powdered with liquid nitrogen and stored at—20 °C, using the protocol published by McKenzie et al. [33] with minor modifications. According to this protocol, leaf tissue (0.25 g) was homogenized with 1.7 mL of a lysis buffer (4 M guanidine isothiocyanate, 0.2 M sodium acetate, 25 mM EDTA, 2.5% PVP-40 and 1% 2-mercaptoethanol) using mortars and pestles or, alternatively, by Tissue Lyser (Qiagen, Hilden, Germany), using beads. Samples were then centrifuged at maximum speed for 6 min; 1 mL of the homogenate was then transferred in a new microcentrifuge tube, supplemented with 100 µL of 10% of N-Lauroylsarcosine sodium salt solution and incubated for 10 min at 70 °C. The homogenate was then poured into the QIAshredder spin columns from RNeasy Plant Mini kit (Qiagen, Hilden, Germany) and total RNA was extracted according to the manufacturer’s instruction of the kit. 

### 4.3. RT-PCR Amplification

To molecularly identify and characterize the three hop *Carlavirus* species, primer pairs able to specifically amplify the CP encoding region (ORF5) of HpMV, HpLV (complete) and AHLV (partial) were employed for the RT-PCR amplification (Table 4). Primer pairs specific for HpMV and HpLV were designed and developed in this study, after multiple alignment of sequences retrieved from the National Center for Biotechnology Information (NCBI) database and tested in silico for their specificity by Primer-BLAST program on the NCBI platform. The primer pair published by Eastwell and Druffel [29] was used for AHLV.

All samples were submitted to specific amplification with the selected primer pairs by a two-step RT-PCR protocol. Specifically, 2 µL of total RNA were added to 18 µL of a prepared RT mixture for random cDNA synthesis, consisting of: 1X reverse transcriptase enzyme buffer (50 mM Tris-HCl pH 7.4, 75 mM KCl, 5 mM MgCl_2_), 4 mM dNTPs, 1 mM DTT, 1 µL of 20 µg Random primers (Promega, Madison, WI, USA) and 5 U reverse transcriptase M-MLV (Invitrogen, Thermofisher scientific, Waltham, MA, USA). Reverse transcription was performed at 42 °C for 45 min, followed by 5 min at 95 °C and 5 min at 4 °C.

For the specific PCR amplification, 2 µL of synthesized cDNA were added to 23 µL of amplification mixture, containing: 1X PCR Master Mix (Promega, Madison, WI, USA) and 0.2 mM of each primer. 

### 4.4. Cloning, Sequencing and Phylogenetic Analysis

To molecularly characterize the identified virus species, amplification products obtained from 12 samples (Table 1) selected among those resulted positives for HpMV (2), HpLV (9) and AHLV (1), were cloned and sequenced.

Amplicons were cleaned by Amicon Ultra-0.5 Centrifugal Filter Units (Merck, Boston, MA, USA), ligated into the pGem-T vector and cloned according to the manual instruction of the pGem-T-Easy vector system (Promega, Madison, WI, USA). Transformed plasmids were isolated by Quantum Prep^®^ Plasmid Miniprep Kit (Bio-Rad, Hercules, CA, USA) and submitted to Sanger sequencing on both ends (Eurofins Genomics, Konstanz, Germany) employing the Sp6 and T7 primers. The obtained sequences were analysed and compared using the web software Clustal Omega (EMBL-EBI) https://www.ebi.ac.uk/Tools/msa/clustalo/ (accessed on 30 August 2023) and Nucleotide BLAST available online at the web page https://blast.ncbi.nlm.nih.gov/Blast.cgi (accessed on 30 August 2023). Nucleotide sequences of the ORF5 coding for the putative CP were then included to phylogenetic analysis (Maximum Likelihood method based on the Kimura 2-parameter model, with a bootstrap phylogenetic test (1000 replicates) performed using MEGA X software [35].

### 4.5. High Throughput Sequencing (HTS) and Bioinformatic Analysis

To further investigate the genome features of detected *Carlavirus* species total RNAs from five selected samples (Table 1) were submitted to HTS for the full genome sequencing of the infecting carlavirus. Specifically, total RNAs extracted as previously described, were sent to Macrogen Europe and used as a template for library preparation using TruSeq Stranded Total RNA LT Sample Prep Kit for Plant (Illumina, San Diego, CA, USA) and sequenced by Illumina platform (Illumina NovaSeq, San Diego, CA, USA). The quality of the raw reads was assessed with the FASTQC software, version 0.11.9, then low quality bases and adapter sequences were trimmed with the BBDuk plugin embedded in Geneious software, version 2021.1 (minimum Phred quality 25 and minimum length 35 bp). The quality of the trimmed reads was checked again with FASTQC. A classification and quantification of the virome composition of the samples was performed with the software GAIA and GAIA 2.0 (Sequentia Biotech, Barcelona, Spain). To assemble the carlavirus genomes the trimmed reads were mapped against the reference sequences (HpLV—NC_002552.1; HMV—NC_010538.1 and AHLV—NC_017859.1) with minimap2 mapper embedded in Geneious software version 2021.1 and then extracted to produce FASTQ files. An assembly was performed for each genome separately using SPAdes assembler and providing the NCBI sequences as references. The obtained contigs were further assembled with CAP3 [36] and then a BLAST filter was applied to remove small and redundant contigs. The assembled sequences were analysed using the ORF Finder tool available on the website http://www.ncbi.nlm.nih.gov/orffinder/ (accessed on 30 August 2023) and compared to the previously characterized genomes using nucleotide or protein BLAST available on the website https://blast.ncbi.nlm.nih.gov/Blast.cgi (accessed on 30 August 2023). Phylogenetic analysis of complete genome sequences was performed using MEGA X software [35]. Maximum Likelihood method based on the Kimura 2-parameter model and General Time Reversible model, with a bootstrap phylogenetic test (1000 replicates) was used.

## Figures and Tables

**Table 1:** Results of the molecular tests performed for the specific detection of HpLV, HpMV and AHLV in the hop samples analyzed. GenBank accession numbers of the nucleotide sequences determined for the selected HpLV, HpMV and AHLV isolates are listed.

Cultivar	Sample Code	Identified Virus (No. Infected Samples/Analysed)	Sequenced Isolates	
			Sanger (Acc. n. ORF5)	HTS (Acc. n. Full Genome)
Chinook Columbus Cascade Hallertauer Magnum Nugget Opal	C1, C2, C3 C4, C5 C6 C10 C11 C12	HpLV (3/3) HpLV (2/2) HpLV HpLV HpLV HpLV		
	
	
	
	
	
Columbus Yeoman	RM4, RM5 RM6, RM7	HpLV (2/2) HpLV (2/2)		
	
Spalter Hallertauer Magnum Spalt Mittlefruh Saaz	RI2 RI3 RI4, RI5 RI6 RI8	HpLV HpLV HpLV (2/2) HpLV HpLV		
	
	
			RI6 (MT251188)	RI6 (ON409683)
	
Cascade Centennial	SUB1, SUB2, SUB3 SUB4	HpLV (3/3) HpLV		
	
Cascade	CA2, CA4, OB1	HpLV (3/3)	OB1 (MT251191)	
Perle	PE	HpLV		
Centennial	CEN1, CEN2, CEN3, CEN5	HpLV (3/4),		CEN2 (ON409680)
		HpMV (2/4)	CEN1 (MT251193) CEN2 (MT251194)	CEN2 (ON409684)
Cascade Nugget Centennial	RN2 TDF1 TDF3	HpLV HpLV HpLV	RN2 (MT251184)	
	
			TDF3 (MT251185)	
Wild hop	RN1	HpMV	RN1 (MT251195)	RN1 (ON409685)
Mounth Hood Centenial Fuggle Sorachi Ace	FZ1 FZ3 FZ4 FZ5	HpLV, AHLV HpLV, AHLV HpLV HpLV		
			FZ3 (MT251183)	FZ3 (ON409681)
			FZ3 (MT251192)	FZ3 (ON409686)
	
			FZ5 (MT251187)	FZ5 (ON409682)
Nugget Hallertauer Magnum Cascade	CS1 CS2 CS3	HpLV HpLV HpLV		
	
	
Saazer Magnum Hersbruker Prima Domus Nugget King Goldwin Fuggle	H1 H2 H3 W1 W2 W3 W4	HpLV HpLV HpLV HpLV HpLV HpLV HpLV	H1 (MT251189)	
	
	
	
			W2 (MT251190)	
	
	
unknown	Abr1	HpLV	Abr1 (MT251186)	
unknown	Ma1	HpLV		
**Commercial cvs.** **Wild hop**		**49 HpLV, 2 HpMV, 2 AHLV** **1 HpMV**		
**Total**		**49/51 HpLV (96.1%), 3/51 HpMV (5.9%), 2/51 AHLV (3.9%)**		

**Table 2:** Results of the BLAST analysis performed on the six sequenced ORFs of each assembled genome; for each isolate the ORF’s length both at nucleotide (nt) and aminoacidic (aa) level and the percentage of identity with sequences retrieved from NCBI database are reported. A heatmap of the identity percentage was created from white (100% identity) to dark gray (low identity).

Isolate	ORF1			ORF2			ORF3			ORF4			ORF5			ORF6		
(Virus)	Lenght	%	ID	Lenght	%	ID	Lenght	%	ID	Lenght	%	ID	Lenght	%	ID	Lenght	%	ID
**FZ3**	5934 nt	**98.06%**	KR185345.1	705 nt	**99.01%**	JQ245696.1	321 nt	**99.38%**	JQ728538.1	201 nt	**98.01%**	KR185345.1	933 nt	**98.07%**	JQ728538.1	345 nt	**98.84%**	KR185345.1
(AHLV)	1977 aa	**98.63%**	YP006297586.1	234 aa	**98.72%**	YP006297587.1	106 aa	**100%**	ALJ56055.1	66 aa	**100%**	ALJ56056.1	310 aa	**98.71%**	ALJ56057.1	114 aa	**98.25%**	AFI61530.1
**CEN2**	5946 nt	**95.44%**	AB032469.1	696 nt	**96.41%**	KP861891.1	327 nt	**96.94%**	AB032469.1	183 nt	**97.81%**	KP861891.1	921 nt	**98.26%**	EF202599.1	315 nt	**97.78%**	KP861891.1
(HpLV)	1981 aa	**97.73%**	NP066258.1	231 aa	**97.04%**	CDK36472.1	108 aa	**99.07%**	NP_066260.1	60 aa	**98.33%**	CDK36474.1	306 aa	**99.35%**	NP066262.1	104 aa	**100%**	AJR19308.1
**FZ3**	5946 nt	**95.14%**	AB032469.1	696 nt	**96.84%**	AB032469.1	327 nt	**96.74%**	AB032469.1	183 nt	**98.36%**	HG793797.1	921 nt	**98.81%**	EF202599.1	315 nt	**97.78%**	KP861891.1
(HpLV)	1981 aa	**97.38%**	NP066258.1	231 aa	**98.27%**	AJR19304.1	108 aa	**99.07%**	NP066260.1	60 aa	**98.33%**	CDK36474.1	306 aa	**99.67%**	ABN68950.1	104 aa	**100%**	AJR19308.1
**FZ5**	5946 nt	**96.82%**	AB032469.1	696 nt	**96.98%**	KP861891.1	327 nt	**95.11%**	AB032469.1	183 nt	**97.81%**	HG793797.1	921 nt	**99.35%**	EF202599.1	315 nt	**97.14%**	KP861891.1
(HpLV)	1981 aa	**98.99%**	NP066258.1	231 aa	**98.27%**	AJR19304.1	108 aa	**97.22%**	NP_066260.1	60 aa	**98.33%**	CDK36474.1	306 aa	**99.67%**	ABN68950.1	104 aa	**98.08%**	AJR19308.1
**RI6**	5946 nt	**97.70%**	KP861891.1	696 nt	**99.14%**	KP861891.1	327 nt	**99.08%**	KP861891.1	183 nt	**98.91%**	KP861891.1	921 nt	**98.91%**	KP861891.1	315 nt	**99.68%**	KP861891.1
(HpLV)	1981 aa	**98.54%**	NP066258.1	231 aa	**100%**	AJR19304.1	108 aa	**100%**	AJR19305.1	60 aa	**100%**	CDK36474.1	306 aa	**100%**	AJR19307.1	104 aa	**100%**	AJR19308.1
**CEN2**	5891 nt	**93.81%**	EU527979.1	689 nt	**94.78%**	EU527979.1	326 nt	**93.58%**	EU527979.1	206 nt	**93.72%**	FJ463807.1	923 nt	**95.56%**	FJ463804.1	308 nt	**97.73%**	FJ463802.1
(HpMV)	1963 aa	**96.99%**	YP001798592.1	229 aa	**97.38%**	YP001798593.1	108 aa	**100%**	YP001798593.1	68 aa	**92.65%**	ACS45270.1	307 aa	**100%**	QKY12192.1	102 aa	**99.02%**	ACS45257.1
**RN1**	5891 nt	**98.93%**	EU527979.1	689 nt	**98.99%**	EU527979.1	326 nt	**99.39%**	EU527979.1	206 nt	**100%**	FJ463810.1	923 nt	**99.13%**	FJ463804.1	308 nt	**99.35%**	FJ463810.1
(HpMV)	1963 aa	**99.08%**	YP001798592.1	229 aa	**99.56%**	YP001798593.1	108 aa	**100%**	YP001798593.1	68 aa	**100%**	YP_001798595.1	307 aa	**100%**	ACS45220.1	102 aa	**99.02%**	YP001798597.1

**Table 3:** Geographical origin and number of samples per cultivar submitted to molecular analyses for identification of the infecting *Carlavirus* species.

Region	Sampling Site	No. of Samples	Cultivar	No. of Samples/Cultivar	Sample Code
Lazio	LAZ1	9	Chinook Columbus Cascade Hallertauer Magnum Nugget Opal	3 2 1 1 1 1	C1, C2, C3 C4, C5 C6 C10 C11 C12
	LAZ2	4	Columbus Yeoman	2 2	RM4, RM5 RM6, RM7
	LAZ3	6	Spalter Hallertauer Magnum Spalt Mittlefruh Saaz	1 1 2 1 1	RI2 RI3 RI4, RI5 RI6 RI8
	LAZ4	4	Cascade Centennial	3 1	SUB1, SUB2, SUB3, SUB4
Tuscany	TOS1	8	Cascade Perle Centennial	3 1 4	CA2, CA4, OB1 PE CEN1, CEN2, CEN3, CEN5
Emilia- Romagna	EMI6	3	Cascade Nugget Centennial	1 1 1	RN2 TDF1 TDF3
		1	Wild hop	1	RN1
	EMI3	4	Mounth Hood Centenial Fuggle Sorachi Ace	1 1 1 1	FZ1 FZ3 FZ4 FZ5
	EMI1	3	Nugget Hallertauer Magnum Cascade	1 1 1	CS1 CS2 CS3
Abruzzo	ABR1	7	Saazer Magnum Hersbruker Prima Domus Nugget King Goldwin Fuggle	1 1 1 1 1 1 1	H1 H2 H3 W1 W2 W3 W4
	ABR2	1	unknown		Abr1
Marche	MAR1	1	unknown		Ma1
**Total**		**50** **1**	**Commercial cvs.** **Wild hop**		
		**51**			

**Table 4:** List of the primer pairs used for the identification and characterization of hop carlaviruses.

Target Virus	Sequence (5′-3′)	Amplicon Size (bp)	Reference
**AHLV**	Rev—TCAGTGCGCTTGTCGAAACTC Fw—ATGTCGAACGTTGAAAGG	931	[34]
**HpLV**	Rev—AGTCAACAGCAAAGCGACAC Fw—AGCAGTAGATGCAAGTTGAAG	1.538	This work
**HpMV**	Rev—AGCACGCCACCAGTGCAT Fw—AAGTCCCTTGGGGGTTGTGG	998	This work

## Data Availability

The datasets generated during and/or analysed during the current study are available from the corresponding author on reasonable request.
